# A quantitative evaluation of gross versus histologic neuroma formation in a rabbit forelimb amputation model: potential implications for the operative treatment and study of neuromas

**DOI:** 10.1186/1749-7221-6-8

**Published:** 2011-10-13

**Authors:** Jason H Ko, Peter S Kim, Kristina D O'Shaughnessy, Xianzhong Ding, Todd A Kuiken, Gregory A Dumanian

**Affiliations:** 1 Department of Surgery, Division of Plastic and Reconstructive Surgery, Northwestern University, Feinberg School of Medicine, Chicago, IL, USA; 2 Department of Pathology, Northwestern University, Feinberg School of Medicine, Chicago, IL, USA; 3 Neural Engineering Center for Artificial Limbs (NECAL), Rehabilitation Institute of Chicago, Chicago, IL, USA

**Keywords:** Neuroma, targeted reinnervation, axon reaction, histomorphometry, brachial plexus

## Abstract

**Background:**

Surgical treatment of neuromas involves excision of neuromas proximally to the level of grossly "normal" fascicles; however, proximal changes at the axonal level may have both functional and therapeutic implications with regard to amputated nerves. In order to better understand the retrograde "zone of injury" that occurs after nerve transection, we investigated the gross and histologic changes in transected nerves using a rabbit forelimb amputation model.

**Methods:**

Four New Zealand White rabbits underwent a forelimb amputation with transection and preservation of the median, radial, and ulnar nerves. After 8 weeks, serial sections of the amputated nerves were then obtained in a distal-to-proximal direction toward the brachial plexus. Quantitative histomorphometric analysis was performed on all nerve specimens.

**Results:**

All nerves demonstrated statistically significant increases in nerve cross-sectional area between treatment and control limbs at the distal nerve end, but these differences were not observed 10 mm more proximal to the neuroma bulb. At the axonal level, an increased number of myelinated fibers were seen at the distal end of all amputated nerves. The number of myelinated fibers progressively decreased in proximal sections, normalizing at 15 mm proximally, or the level of the brachial plexus. The cross-sectional area of myelinated fibers was significantly decreased in all sections of the treatment nerves, indicating that atrophic axonal changes proceed proximally at least to the level of the brachial plexus.

**Conclusions:**

Morphologic changes at the axonal level extend beyond the region of gross neuroma formation in a distal-to-proximal fashion after nerve transection. This discrepancy between gross and histologic neuromas signifies the need for improved standardization among neuroma models, while also providing a fresh perspective on how we should view neuromas during peripheral nerve surgery.

## Background

When a peripheral nerve is transected, the distal nerve segment undergoes Wallerian degeneration and, without coaptation to proximal nerve tissue, eventually disappears [1]. The proximal nerve stump, in contradistinction, has the ability to regenerate and send axon sprouts into the distal nerve segment, potentially proceeding to the target organs [2,3]. However, when regenerating axons fail to reach the distal segment, a neuroma forms, and axons cease to grow [4]. On a microscopic level, these neuromas consist of disorganized, chaotic myelinated axons encased in significant connective tissue stroma [5], and they are frequently sensitive to pressure, causing a classic focal neuroma pain [6,7]. Neuroma pain can be both physically and psychologically disabling and is often difficult to treat [8,9]. Numerous surgical techniques have been proposed for the prevention and treatment of neuromas, including simple ligation [10,11]; capping the nerve stump with various materials [12-15]; translocation into nerve tissue through end-to-side or centro-central coaptation [16-18]; and transposition of the nerve ending into bone [8,19], fat [20,21], muscle [6,22-24], and, more recently, vein [25-28]. As implied by the large number of techniques to prevent and treat neuromas, there is no consensus yet on which method is most effective. Regardless of technique, however, the fundamental principle of neuroma surgery involves excising the injured nerve segment proximally to the level of grossly normal fascicles. Yet the zone of injury of a peripheral nerve ending in a classic neuroma is not defined, and understanding the microanatomy of these situations is important both in clinical peripheral nerve surgery, as well as for the standardization of all animal nerve models that attempt to investigate neuroma treatments.

Targeted reinnervation is a revolutionary strategy performed in upper extremity amputees where the stumps of amputated nerves of the brachial plexus are transferred to denervated, otherwise functionless, remnant muscles in the shoulder, chest, and/or proximal arm, in order to achieve a functioning neural-machine interface that allows amputees to voluntarily control motorized prostheses just as they would control their native limbs [29-34]. In order to further investigate targeted reinnervation at a level just distal to the brachial plexus, we developed a novel rabbit forelimb amputation model that is a well-tolerated and reproducible quantitative model of end-neuroma formation [35]. An amputation model was created to better simulate the clinical scenario of limb amputation, as well as to increase the number of neuromas created per animal (and thereby decrease the total number of animals sacrificed), and the amputation was performed in the proximal forelimb in order to mimic the clinical scenario that is often encountered in targeted reinnervation. Although previous studies have examined the retrograde axonal changes that occur after nerve transaction [36-42], there is sparse data regarding the distal-to-proximal histologic changes that occur in the proximal nerve stump, as they relate to gross nerve appearance, after amputation injury at the brachial plexus level.

## Materials and methods

This study was approved by the Northwestern University Institutional Animal Care and Use Committee (IACUC) prior to its initiation. Four 6-month old (2.5-3.5 kg) female New Zealand White rabbits (Covance Inc., Princeton, NJ) were acquired and single-housed with food and water *ad libitum*.

### Operative Technique

**Figure 1:**
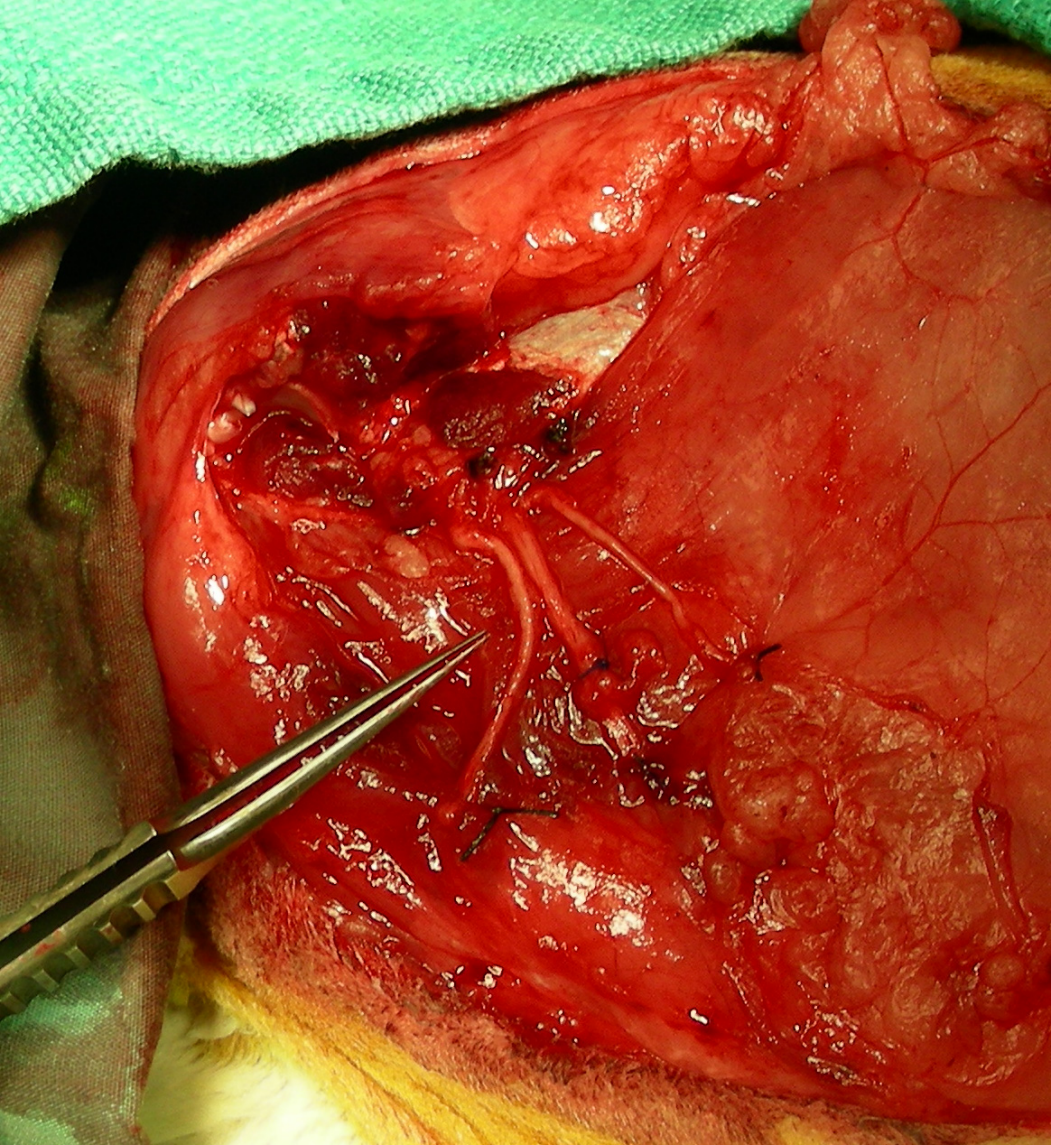
**The amputated stumps of the median (*left*), radial (*center*), and ulnar (*right*) nerves are loosely sutured to the pectoralis superficialis transversus using 7-0 polypropylene suture to ease identification and location of the neuromas at the time of harvest**.

The pre- and post-operative care of the animals were outlined in detail in a previous study, as was the surgical technique [35]. Briefly, under sterile conditions, an elliptical incision was made around the left proximal forelimb, and the distal skin overlying the forelimb was elevated in a circumferential, de-gloving fashion. The nervous structures--with special attention directed to the median, radial, and ulnar nerves--were exposed and identified as they exited the brachial plexus, and the median, radial, and ulnar nerves were each transected 2 cm distal to where they branched off of the brachial plexus and loosely sutured to the anterolateral aspect of the normally innervated pectoralis superficialis transversus muscle using 7-0 polypropylene suture (Prolene suture, Ethicon Inc., Somerville, NJ) (Figure 1). All muscles and tendons were disinserted from the humerus, and a shoulder disarticulation amputation was performed. The remaining muscles were sutured together over the glenoid fossa and any remaining bony prominences using 4-0 polyglactin (Vicryl suture, Ethicon), and the skin incision was closed in a running subcuticular fashion using 4-0 polyglactin suture. Following recovery, the rabbits were inspected daily for abnormal activity, evidence of pain, and post-operative wound complications.

### Tissue Harvest and Preparation

**Figure 2:**
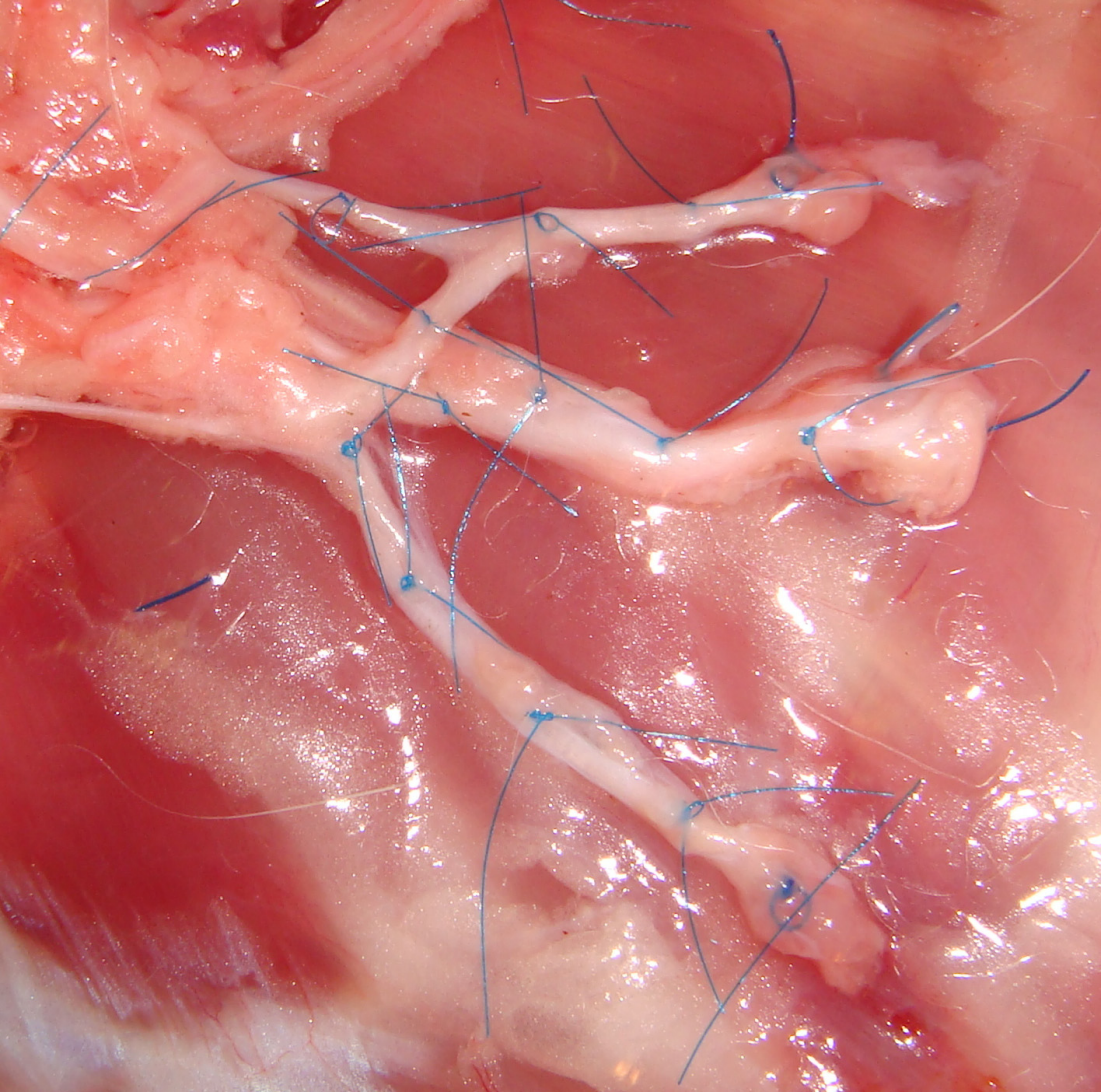
**Six to 8 weeks post-amputation, after the distal 5 mm of the median (*top*), radial (*center*), and ulnar (*bottom*) neuromas was excised, 7-0 polypropylene sutures were placed at the distal segment, and at 5, 10, and 15 mm proximally toward their branch points off the brachial plexus**.

Eight weeks post-amputation, the rabbits were euthanized, and the original surgical incision was re-opened, with the median, radial, and ulnar neuromas dissected out and brought to length. After excising the distal 5 mm of neuroma/nerve, which is typically performed in targeted reinnervation procedures, in addition to other nerve transfer and neuroma procedures, 7-0 polypropylene sutures were used to mark the remaining distal segment of each nerve, in addition to 5 mm proximally, 10 mm proximally, and 15 mm proximally toward their branch points off the brachial plexus (Figure 2). Serial nerve sections were harvested at each location as indicated by the suture markings. In the contralateral limb, serial nerve sections were obtained from the median, radial, and ulnar nerves at corresponding lengths--distal end, 5 mm proximally, 10 mm proximally, and 15 mm proximally--relative to their branch points off the brachial plexus to serve as controls. In all animals, after excising the distal 5 mm of nerve tissue, 20 mm proximally represented the level of the brachial plexus.

Harvested nerve specimens (n = 96 total) were fixed in 4% EM grade glutaraldehyde (Polysciences Inc., Warrington, PA) at 4°C, post-fixed with 2% osmium tetroxide (Polysciences) and serially dehydrated in ethanol. Specimens were embedded in Poly/Bed^® ^812 BDMA (Polysciences) and cut into 1-μm cross-sections with a Leica Ultracut UCT ultramicrotome (Leica Microsystems Ltd., Wetzlar, Germany). Sections were then stained with 1% toluidine blue, and mounted and cover-slipped for imaging.

### Histomorphometric Analysis

A Nikon DS-5M-U1 (Nikon Instruments Inc., Melville, NY) digitizing camera was mounted onto a Nikon Eclipse 50i (Nikon) microscope with a manually controlled stage. Nikon NIS-Elements BR 2.3 (Nikon) imaging software was used to perform nerve histomorphometric analysis of all slides. Using a semi-automated technique, characterized by dynamic thresholding and manual fiber elimination, [43,44] each nerve was analyzed to determine the nerve cross-sectional area, the myelinated axon count in each nerve cross-section, and the cross-sectional areas of the axons including their myelin sheaths. In order to prevent grading bias, prepared slides from amputated and control sides were randomly assigned numbers for analysis with their identification marks covered.

### Statistical Analysis

Control nerve sections at each location (distal end, 5 mm proximally, 10 mm proximally, and 15 mm proximally) were grouped according to nerve (median, radial, and ulnar nerves), and an analysis of variance (ANOVA) with Bonferroni post-test analysis was performed for each of the three following histomorphometric parameters: 1) nerve cross-sectional area; 2) myelinated axon count; and 3) myelinated axon cross-sectional area. There were no significant differences amongst nerve type for each variable, so the treatment nerves for the median, radial, and ulnar nerves at each location were compared to grouped control nerves for each nerve type using the two-tailed Student's *t*-test to analyze nerve cross-sectional area, myelinated axon count, and myelinated cross-sectional area. A *p*-value < 0.05 was considered statistically significant.

## Results

**Figure 3:**
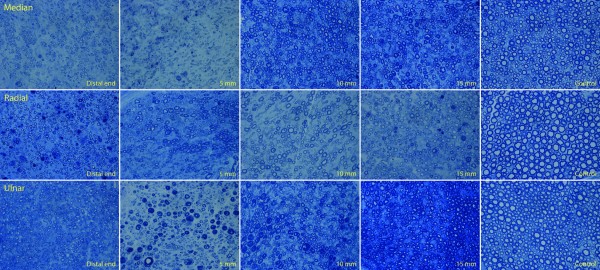
**(*Above*) Median nerve**. (*Center*) Radial nerve. (*Below*) Ulnar nerve with toluidine blue staining at 400× magnification. (*First column*) Smaller, disorganized myelinated fibers, with qualitatively increased amounts of myelin infolding, crenation, and debris are seen at the distal end of each proximal nerve stump. Regenerative clusters with axon sprouting are more prevalent at the distal ends, as is the amount of connective tissue stroma. (*Second, third, and fourth columns*) The myelinated fibers become progressively more organized and larger at 5, 10, and 15 mm proximally, although myelin debris and crenation are still noted. (*Fifth column*) The control nerves demonstrate organized, circular, and larger fibers with no noticeable myelin debris.

Gross examination of the amputated nerve stumps revealed traumatic neuroma tissue that was enlarged with nodular fusiform formation at the distal end of each of the transected nerves. Fibrosis was also present, resulting in adhesions to the surrounding tissue. The aforementioned macroscopic findings, especially the nerve calibers, normalized by 5 mm proximally in all of the transected nerves, and sectioning of the nerves demonstrated grossly normal fascicles 5 mm proximal to the distal end. Microscopically, the nerve architecture at the amputation site was disorganized with extensive nerve fiber regeneration and disorientation. Uneven distribution of regenerative nerve fibers was observed with variation of axonal bundle density from area to area, and marked variation in shape and size of axonal bundles was also observed. Dramatic fibrosis was seen between the regenerative nerve bundles (Figure 3). Under higher microscopic magnification, interstitial stroma between regenerative axonal bundles was fibrotic with collagen deposition. Smaller, disorganized myelinated fibers, with qualitatively increased amounts of myelin infolding, crenation, and debris were seen at the distal end of each proximal nerve stump. In the amputated nerve stumps, axonal regeneration, axonal bundle disorganization and disorientation, and interstitial fibrosis progressively normalized in a distal-to-proximal fashion but are still present even at a distance of 15 mm proximal to the distal neuroma end when compared to control nerve specimens. The aforementioned qualitative observations were confirmed by histomorphometric analysis.

### Nerve Cross-Sectional Area

**Figure 4:**
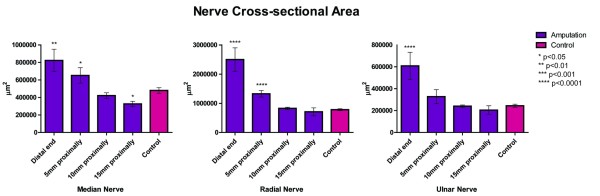
**The nerve cross-sectional area of the median, radial, and ulnar nerves compared to control nerves at the time of harvest (6-8 weeks)**.

As Figure 4 demonstrates, the mean cross-sectional area of the median nerve at the distal end for the amputation group had a 1.7-fold increase compared to that for the control group (p = 0.001), and the median nerve segments at 5 mm were 1.4 times larger than corresponding controls (p = 0.04). Of note, the median nerve sample 15 mm proximally demonstrated a 33% decrease in mean cross-sectional area for the amputation group compared to the control group (p = 0.03). For the radial nerve, the mean cross-sectional area at the distal end was 3.2 times greater in the amputation group than in the control group (p < 0.0001), and at 5 mm proximally, the cross-sectional area for the amputated radial nerve was significantly greater (by a factor of 1.7) compared to control (p < 0.0001). The amputation group demonstrated a 2.5-fold increase in cross-sectional area of the ulnar nerve at the distal end compared to control (p < 0.0001). Once again, the amputated group had a larger mean cross-sectional area at 5 mm proximally, but this was not statistically different than the control group.

### Myelinated Axon Count

**Figure 5:**
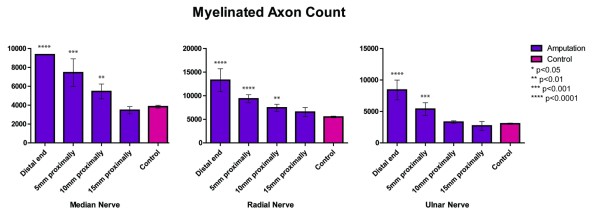
**The myelinated axon count of the median, radial, and ulnar nerves compared to control nerves at the time of harvest (6-8 weeks)**.

As demonstrated in Figure 5, the myelinated axon count at the distal end of the median nerve demonstrated a 2.4-fold increase in the amputation group when compared to the control group (p < 0.0001). Five mm proximally, the axon counts were 1.9 times higher in the amputated nerves (p = 0.0003), and 10 mm proximally, the axon counts were 1.4 times higher in the amputated nerves (p = 0.004). The mean myelinated axon count for the radial nerve was 2.4 times higher at the distal end in the amputation group (p < 0.0001); 1.7 times higher 5 mm proximally in the amputation group (p < 0.0001); and 1.4 times higher 10 mm proximally in the amputation group (p = 0.001). The ulnar nerve demonstrated the same trend with an increased myelinated axon count by a factor of 2.8 for the amputation group at the distal end (p < 0.0001) and a 1.8-fold increase in the amputation group 5 mm proximally (p = 0.005). There was no significant difference in the axon counts for any amputated nerve groups 15 mm proximally compared to the normal control group.

### Myelinated Axon Cross-Sectional Area

**Figure 6:**
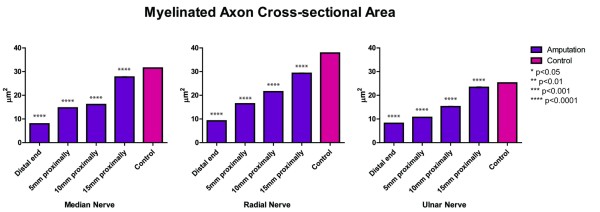
**The myelinated axon cross-sectional area of the median, radial, and ulnar nerves compared to control nerves at the time of harvest (6-8 weeks)**.

Figure 6 shows significant decreases in mean myelinated axon cross-sectional area for the median, radial, and ulnar nerves in amputation versus control groups at all nerve distances (p < 0.0001 for distal end, 5, 10, and 15 mm proximally). The average cross-sectional areas were smallest near the neuroma, and axon cross-sectional areas increased progressively as the nerve was sectioned more proximally. However, the myelinated axon area did not normalize to the control group values. This pattern was also consistently demonstrated in the radial nerve (p < 0.0001 for distal end, 5, 10, and 15 mm proximally) and in the ulnar nerve (p < 0.0001 for distal end, 5, 10, and 15 mm group).

## Discussion

Inspired by findings both in the laboratory and in the operating room, this study was undertaken to better understand the microanatomic changes that occur to the proximal end of a chronically transected peripheral nerve. First described by Waller in 1850 [1], the changes that occur in the *distal *segment of a transected nerve are accordingly referred to as *Wallerian degeneration*; however, in addition to changes in the distal nerve segment, Waller also described the generation of neural tissue from the *proximal *nerve, which was further described and pioneered by Ramón y Cajal [2].

In the proximal nerve segment, a series of histologic changes occur in a process referred to as the axon reaction, retrograde effect, and/or traumatic degeneration, amongst other names [45-47]. During the axon reaction, according to Sunderland, anywhere from 17 to 94% of nerve fibers die [48], mostly as a result of diminished target-derived neurotrophic support [49,50]. In several studies on the axon reaction in a cat hindlimb amputation model, Dyck et al. described the series of cellular events after permanent axotomy as they progress from axonal atrophy to demyelination and, ultimately, axonal degeneration [37,38,51]. These changes begin, and are more severe, distally but also affect more proximal segments of peripheral nerve, with the traumatic axotomy initiating the cellular changes in a distal-to-proximal fashion [51]. In order to evaluate the gross and histologic changes that occur to the entire nerve stump after nerve transection, we used the rabbit forelimb amputation model previously developed in our laboratory to analyze serial nerve sections obtained in a distal-to-proximal fashion from the distal neuroma to the level of the brachial plexus--a clinical scenario often seen in targeted reinnervation patients.

In this study, significant increases in nerve cross-sectional area and myelinated axon count between treatment and control limbs were demonstrated at the distal nerve ends, consistent with previous studies [35]. Given what is known about neuroma histology, the increased nerve cross-sectional areas of the distal nerve endings in our study are mostly due to increased amounts of connective tissue stroma and inflammation in response to injury [5]. The increased myelinated axon counts in the distal nerve sections seen in our study can be explained by the fact that after peripheral nerve transection, a single parent axon produces numerous daughter sprouts [52-54]. As demonstrated in Table 1, the total myelinated axon area accounts for 25-32% of the total nerve cross-sectional area for both the treatment nerves 15 mm proximally and the control nerves. However, this ratio progressively decreases to only 5-11% of total nerve area when moving distally down the nerve, even though the number of myelinated axon fibers increases. Although the differences in total axon area and nerve cross-sectional area seen distally are partly due to increased connective tissue and inflammation, there is also less myelinated tissue distally, which may be due to axon demyelination, and thus the true count of axon sprouts--myelinated and unmyelinated--would be even higher than measured in this study. Additionally, the cross-sectional area of myelinated axons was significantly decreased in all serial sections of the treatment nerves, indicating that, without a distal target for these sprouts to grow into, axonal atrophy continued to proceed in a distal-to-proximal fashion to the level of the brachial plexus. However, increases in nerve cross-sectional area and myelinated axon count diminished distally-to-proximally with values normalizing by 15 mm proximal to the amputation. With time and increased axon loss, the amputated nerves may reduce in size even further. For example, the cross-sectional area of the median nerve at the point 15 mm proximally was significantly decreased compared to that of the control nerve.

**Table 1:** Measurements of Total Axon Area and Nerve Area

Nerve	Nerve cross-sectional area (μm^2^) (Normalized)	Myelinated axon count (Normalized)	Myelinated axon cross-sectional area (μm^2^) (Normalized)	Total myelinated axon area (μm^2^)*	Total myelinated axon area/Nerve cross-sectional area
**Median**					
Distal end	823100 (1.71)	9349 (2.44)	8.0 (0.25)	74325	0.090
5 mm proximally	651600 (1.36)	7434 (1.94)	14.7 (0.47)	109205	0.168
10 mm proximally	421600 (0.88)	5449 (1.42)	16.1 (0.51)	87729	0.208
15 mm proximally	326000 (0.68)	3463 (0.90)	27.7 (0.88)	95925	0.294
Control	480300 (1.00)	3839 (1.00)	31.5 (1.00)	120967	0.252
**Radial**					
Distal end	2498000 (3.19)	13280 (2.42)	9.2 (0.24)	121817	0.049
5 mm proximally	1327000 (1.70)	9346 (1.70)	16.4 (0.43)	153461	0.116
10 mm proximally	825100 (1.05)	7445 (1.36)	21.5 (0.57)	160216	0.194
15 mm proximally	708100 (0.91)	6525 (1.19)	29.3 (0.77)	191052	0.270
Control	782300 (1.00)	5494 (1.00)	37.9 (1.00)	207948	0.266
**Ulnar**					
Distal end	608500 (2.49)	8407 (2.76)	8.1 (0.32)	68391	0.112
5 mm proximally	327400 (1.34)	5370 (1.76)	10.7 (0.42)	57459	0.176
10 mm proximally	240500 (0.98)	3308 (1.08)	15.2 (0.60)	50381	0.209
15 mm proximally	204600 (0.84)	2701 (0.89)	23.4 (0.93)	63122	0.309
Control	244500 (1.00)	3050 (1.00)	25.2 (1.00)	76952	0.315

*Total myelinated axon area = Myelinated axon count × Myelinated axon cross-sectional area

In a rabbit peroneal nerve injury model, Gutmann and Sanders demonstrated that myelinated fiber sizes were significantly smaller 15 mm proximal to the lesion compared to controls up to 130 days after injury, with only slightly increased myelinated fiber numbers [41]. Our findings are more consistent with those of Aitken, who demonstrated that in the nerve to the gastrocnemius muscle of the rabbit, the number of myelinated fibers proximal to a neuroma increased by greater than 50% after nerve transection, with an elevated number of small myelinated fibers [55]. However, although Aitken noted that the marked increase in myelinated fibers occurred immediately proximal to neuromas, how far proximally the regenerating fibers grew in a retrograde fashion was not evaluated. Using a mouse sural nerve model, Scadding and Thomas demonstrated a 37% increase in myelinated axons at a distance of 1.5 cm proximal to the point of nerve section after 10 weeks [56]. In our study, the increased number of myelinated axons in the amputated nerves progressively normalized compared to controls in a distal-to-proximal fashion; therefore, there were no significant differences in the median, radial, and ulnar neuromas in terms of myelinated axon counts at a distance of 15 mm proximally. However, it is important to note that whereas Scadding and Thomas used a mouse sural (purely sensory) nerve model, our study employed larger caliber mixed (motor and sensory) nerves in the rabbit, making comparisons difficult to draw. In addition, unlike the methodology of Scadding and Thomas, the distal 5 mm of neuroma was excised and excluded for each nerve in our study in an effort to replicate what is done in targeted reinnervation procedures, thereby making the "15 mm proximal" group in our study, in reality, 20 mm from the distal end of the neuroma.

The extent of retrograde degeneration of amputated nerves has both functional and therapeutic implications since aberrant discharges are spontaneously generated by both neuromas and retrograde axon sprouts [54,57-61]. In a rat sciatic nerve model, Wall and Gutnick demonstrated that smaller fibers within neuromas produce ongoing spontaneous activity that may be responsible for sensations of pain [61]. In a study assessing neuromas of the superficial radial nerve in baboons, Meyer et al. found that spontaneously active fibers were present in the neuromas, consisting of both myelinated and unmyelinated axons that were mechanically sensitive, with apparent crosstalk between fibers within the neuroma [7]. Sixty-seven percent of the spontaneously active fibers in the neuroma were unmyelinated, compared to 19% in the control, pointing out a potential link between neuromas and nociceptive pathways. Amir and Devor showed in a rat sciatic neuroma model that spontaneous discharges occurred in afferents that terminated in the neuroma, as well as in afferents that had emitted retrograde sprouts [57]. In fact 39% of fibers with retrograde sprouting carried spontaneous ongoing discharges, and, conversely, the authors point out those axons with spontaneous activity were significantly more likely to have a retrograde sprout. Amir and Devor proposed that individual neurons that emit retrograde sprouts have an unusually high likelihood of firing spontaneously [57], which, in conjunction with an increased capacity for myelinated A-β sprouts to make contact with nociceptive-specific neurons [62-64], can result in pain.

Repeated noxious stimuli--as in the case of an acutely injured peripheral nerve, in addition to spontaneous discharges from neuromas and sprouting axons--lead to decreased activation thresholds, and responses to subsequent stimuli are thereby amplified [65,66]. The aforementioned increase in excitability further exacerbates nociception by leading to decreased inhibition from afferent fibers [67-69], thereby creating a state of central sensitization of neural tissue involved in pain perception. Whereas potential therapies for central pain pathways are beyond the scope of this discussion [70-72], the noxious stimuli in the peripheral nervous system that ignite the cycle of events that ultimately lead to central perceptions of pain are important for this discussion. With retrograde sprouts being able--and more likely--to produce spontaneous, ectopic discharges after peripheral nerve injury, it is possible that neuroma treatment procedures should focus not only on excising the neuromas, but also on removing any proximal neural tissue that contains retrograde axonal sprouts.

During clinical procedures for the treatment of symptomatic neuromas, in addition to nerve transfer procedures like targeted reinnervation, complete excision of the "neuroma" is recommended, but where exactly does the neuroma begin? In our study, gross neuroma appearance did not correlate with the "zone of injury" of the proximal nerve stump on an axonal level. Morphologic changes at the axonal level extended beyond the region of gross neuroma formation, measured as nerve cross-sectional area, in a distal-to-proximal fashion after nerve transection, supporting the first of two main intra-operative concepts: First, a normal-sized nerve end does not necessarily mean that the nerve is internally normal. Second, approximately 2 centimeters proximal to a neuroma bulb, in a rabbit, the majority of sprouted axons would be removed. Given the potential for retrograde axon sprouts to produce ectopic, spontaneous, and painful discharges, we propose that cutting back more proximally on the nerve stump, beyond the appearance of grossly normal-appearing fascicles, may be beneficial during neuroma surgery in symptomatic patients. Employing the use of intra-operative frozen sections would be an effective method of minimizing, if not eliminating, any neural tissue that contains retrograde sprouts. However, this raises the interesting question of whether there is an optimal site to cut back on a neuroma that is going to be used for nerve transfer, such as targeted reinnervation. Cutting back further proximally will leave a nerve segment with fewer axon sprouts than using a nerve segment that is closer to the neuroma bulb, though it has yet to be determined whether cutting back in this fashion would have any detrimental functional consequences. Also, there are clinical scenarios for which nerve length is a major limiting factor where it would be unfeasible--detrimental even--to cut back neuromas more proximally, such as when treating neuromas-in-continuity for brachial plexus reconstruction. Injuries to the brachial plexus itself can potentially demonstrate histologic changes proximally to the level of the cervical root or spinal cord, making excision and subsequent reconstruction impractical. On the other hand, when performing targeted reinnervation, the nerves can be cut far proximally (3-12 cm) from the end-neuroma without difficulty or consequence. Therefore, the surgeon must decide how far proximally to cut back on a neuroma based on the clinical indication and overall operative plan.

When considering the discrepancy that exists between gross and histologic neuromas, one must change how we evaluate neuromas, not only clinically, but also with respect to bench research. There is a need for improved standardization among neuroma models in terms of where along the length of the proximal nerve stump measurements should be made. A look at several large-animal neuroma models makes it apparent that little mention is made as to where exactly, whether in the neuroma itself or at a specified distance proximal to the gross neuroma, histologic analysis is being performed [6,73,74]. A neuroma at its largest diameter has different characteristics than a nerve segment just 5 mm proximally, as reinforced by our study. It is imperative that data collection in animal models that relies on axon counts, axon size, and other quantitative parameters must therefore standardize the sites where nerve measurements are made.

## Conclusions

Using a rabbit forelimb amputation model that was developed to further assess targeted reinnervation, we determined that morphologic changes at the axonal level extend beyond the region of gross neuroma formation in a distal-to-proximal fashion after nerve transection at the level of the brachial plexus. Normal-sized nerves do not correlate with normal nerve histomorphometry in this model, and the discrepancy between gross and histologic neuromas indicates potential implications for how neuromas should be viewed, both in the laboratory and in the operating room.

## Competing interests

The authors declare that they have no competing interests.

## Authors' contributions

JK participated in design and execution of the model, histomorphometric analysis, and drafting of the manuscript. PK participated in preparation of the manuscript; KO engineered the imbedding and histomorphometric techniques specific for the needs of this model; XD performed critical macroscopic and microscopic analysis of the histologic specimens; and TK and GD participated in the design and coordination of the model. All authors read and approved the final manuscript.
